# The Framework of Host Innate and Adaptive Immunological Pathways

**DOI:** 10.3390/biomedicines14071630

**Published:** 2026-07-20

**Authors:** Wan-Chung Hu

**Affiliations:** 1 Department of Medical Research, Taipei Tzu Chi Hospital, Buddhist Tzu Chi Medical Foundation, New Taipei City 231, Taiwan; wanchung.hu09@gmail.com or wanchung.hu09@tzuchi.com.tw; 2 Department of Clinical Pathology, Taipei Tzu Chi Hospital, Buddhist Tzu Chi Medical Foundation, New Taipei City 231, Taiwan; 3 Department of Biotechnology, Ming Chuan University, Taoyuan City 333, Taiwan

**Keywords:** innate immunity, adaptive immunity, γδ T cells, interleukin-35, interleukin-32

## Abstract

**Background**: Host immune responses can be broadly divided into innate and adaptive immunity. Numerous adaptive immune responses have been identified, including TH1, TH2, TH3, TH9, TH17, and TH22 immunity. Within innate immunity, Vγ9-chain γδ T cells are among the most extensively studied immune-cell populations. **Knowledge Gap**: However, the precise functional classification of these innate and adaptive immunological pathways in responses to different types of pathogens remains incompletely understood. **Purpose of the Review**: This review proposes an integrated framework for the detailed functional classification of host innate and adaptive immunological pathways. **Proposed Framework**: Within innate immunity, γδ T cells can be categorized into several functional groups. The clonal anergy and tolerance pathway is associated with Vγ2-chain γδ T cells. The host innate immunological pathway against viruses is associated with Vγ8-chain γδ T cells, whereas the pathway against intracellular microorganisms is associated with Vγ9-chain γδ T cells. The pathway against extracellular microorganisms is associated with Vγ4-chain γδ T cells, the pathway against helminths with Vγ5-chain γδ T cells, and the pathway against insects with Vγ3-chain γδ T cells. Within adaptive immunity, five eradicable immune reactions and four tolerable immune reactions are described. Among the tolerable immune reactions, TH3 is associated with interleukin-35-producing CD4 T cells, whereas TH4 is associated with interleukin-32-producing CD4 T cells. **Significance**: A more precise functional classification of innate and adaptive immunological pathways may provide a useful conceptual basis for combating infections and hypersensitivity disorders.

## 1. Introduction: Innate Immunity

### 1.1. Overview of Host Innate Immunity and Adaptive Immunity

The immune system is a highly coordinated biological network that protects the host from pathogenic microorganisms while preserving tolerance to self-components. This system is classically divided into two interdependent arms: innate immunity and adaptive immunity. Together, these two branches provide immediate defense and long-term, antigen-specific protection, forming an integrated and dynamic host defense system. Understanding the distinct characteristics and cooperative functions of innate and adaptive immunity is essential for elucidating immune homeostasis, host–pathogen interactions, and the mechanisms underlying immune-mediated diseases.

Innate immunity constitutes the first line of defense against invading pathogens and is evolutionarily conserved across multicellular organisms. It relies on germline-encoded receptors, known as pattern recognition receptors, that detect conserved molecular structures shared by broad classes of microorganisms, referred to as pathogen-associated molecular patterns, as well as danger-associated molecular patterns released from damaged host cells. Innate immune responses are rapid, occurring within minutes to hours following pathogen exposure, and involve physical barriers such as the skin and mucosal surfaces, as well as cellular and humoral components including macrophages, neutrophils, dendritic cells, natural killer cells, and the complement system. Although innate immunity lacks antigen specificity and immunological memory, it plays a crucial role in containing early infection, shaping tissue inflammation, and instructing subsequent adaptive immune responses through cytokine production and antigen presentation [1,2].

Adaptive immunity, in contrast, is characterized by antigen specificity, clonal expansion, and immunological memory. This branch of the immune system is mediated primarily by T lymphocytes and B lymphocytes, which express highly diverse antigen receptors generated through somatic gene rearrangement. Adaptive immune responses are slower to develop during initial antigen exposure, typically requiring several days; however, they provide highly specific and potent effector functions, including cytotoxic T-cell-mediated killing of infected cells and antibody-mediated neutralization of extracellular pathogens. A defining feature of adaptive immunity is its ability to generate long-lived memory cells, which confer enhanced protection upon subsequent encounters with the same antigen, forming the basis of vaccination [3].

Despite their distinct properties, innate and adaptive immunity are not independent systems but function in close coordination. Innate immune cells, particularly dendritic cells, serve as critical intermediaries by capturing antigens and presenting them to naïve T cells, thereby initiating adaptive immune responses. Conversely, adaptive immune cells can modulate innate immunity through cytokine secretion and antibody-mediated opsonization, enhancing pathogen clearance. This bidirectional communication ensures an appropriate immune response tailored to the nature, location, and persistence of the antigenic challenge [4].

Dysregulation of either innate or adaptive immunity can result in pathological conditions, including chronic inflammation, autoimmunity, immunodeficiency, and cancer. Consequently, a comprehensive understanding of both immune arms and their regulatory mechanisms is fundamental for advancing immunological research and developing targeted therapeutic strategies. This review aims to synthesize current knowledge on innate and adaptive immune pathways, emphasizing their functional integration and relevance to immune tolerance and disease pathogenesis.

### 1.2. Mechanism of Clonal Anergy of Host Immune Regulation

The immune system must discriminate between self-antigens and foreign antigens to maintain host integrity while providing effective defense against pathogens. Under physiological conditions, immune cells that encounter self-antigens do not elicit immune responses, thereby preventing autoimmunity. A central principle underlying this specificity is the clonal selection mechanism, whereby each individual T or B lymphocyte expresses a unique antigen receptor and is therefore capable of recognizing only a single antigenic determinant. When a clonally distinct T or B cell recognizes a self-antigen, it enters a state of functional unresponsiveness known as clonal anergy, characterized by the absence of effector immune responses.

Clonal anergy is mediated through distinct cellular mechanisms involving γδ T cells and IgD-expressing B cells. γδ T cells arise earlier during thymic development than conventional αβ T cells. TRGV2 γδ T cells encode TCR γδ chains at the first chromosomal location; therefore, TRGV2 γδ T cells may be responsible for clonal anergy. This temporal distinction has important implications for immune tolerance. When a developing clonal T cell encounters a self-antigen, particularly a protein antigen, it preferentially differentiates into a TRGV2 γδ T cell. Consequently, the subsequent αβ T-cell repertoire is selectively shaped to avoid recognition of self-antigens, thereby preserving immune tolerance. Recognition of self-antigens by TRGV2 γδ T cells results in clonal anergy rather than immune activation, reinforcing central and peripheral tolerance mechanisms. Other γδ T cells are responsible for innate immunity, which should respond earlier than adaptive αβ T cells [5,6].

An analogous process operates within the B-cell compartment. Mature B lymphocytes co-express immunoglobulin D (IgD) and immunoglobulin M (IgM) on their cell surface. Antigen recognition through IgD serves a tolerogenic function: when IgD binds self-antigens, B cells undergo clonal anergy without initiating antibody-mediated immune responses. In contrast, recognition of foreign antigens through surface IgM triggers B-cell activation, leading to proliferation and differentiation. Activated IgM-bearing B cells subsequently undergo immunoglobulin class-switch recombination to generate IgG, IgE, or IgA antibodies, enabling effective humoral immunity against pathogens. This functional dichotomy between IgD- and IgM-mediated signaling highlights the critical role of IgD in maintaining B-cell tolerance [7,8].

Experimental evidence supports the tolerogenic roles of certain γδ T cells and IgD-expressing B cells. Multiple studies have demonstrated that IgD administration ameliorates autoimmune arthritis in animal models, suggesting a direct immunoregulatory effect. Similarly, certain γδ T cells have been shown to protect against graft-versus-host disease following organ transplantation, further underscoring their importance in immune tolerance [9,10].

γδ T cells are heterogeneous and can be subdivided according to δ-chain usage and tissue distribution. γδ1 T cells are predominantly localized in the intestinal mucosa, where they contribute to clonal anergy toward dietary antigens and innate immunity against pathogens in the gastrointestinal tract. This mechanism underlies oral tolerance and explains why common food proteins typically do not provoke immune responses. γδ2 T cells circulate mainly in peripheral blood and are primarily responsible for inducing tolerance to systemic self-antigens and mediating innate immunity against pathogens in the bloodstream. γδ3 T cells are enriched in the liver, an organ with intrinsic immune-tolerant properties and a central role in protein metabolism. These cells are essential for maintaining hepatic immune tolerance, particularly toward endogenous and metabolized antigens. γδ3 T cells are also responsible for innate immunity against pathogens entering the hepatosplenic circulation [9]. Overall, the previously proposed framework of host immunity requires further updating [11,12].

### 1.3. Framework of Innate Immunological Pathways

Based on the eradicable host adaptive immune responses described above, five immunological pathways can be identified: TH1, TH2a, TH2b, TH22, and THαβ. γδ T cells play vital roles in mediating innate immune reactions. There are five major types of γ chains and three major types of δ chains in γδ T cells. In our previous publication, we proposed that the three δ-chain subtypes of γδ T cells are related to the organ and tissue locations of these cells. δ1 γδ T cells are found in the intestine or skin, δ2 γδ T cells are found in the blood, and δ3 γδ T cells are found in the liver [13]. The functions of the γ-chain subtypes may be associated with host immune responses against different types of pathogens. Excluding pseudogenes, humans have six γ-chain subtypes, including Vγ2, Vγ3, Vγ4, Vγ5, Vγ8, and Vγ9. Thus, five or six innate immunological pathways can be described, as outlined below.

### 1.4. The Clonal Anergy and Tolerance Pathway Is Associated with Vγ2-Chain γδ T Cells

The γδ T cells associated with clonal anergy are proposed to be Vγ2-chain T cells. The Vγ2 chain appears first in the chromosomal γ-chain sequence; therefore, it is reasonable to propose an association between the Vγ2 chain and clonal anergy. Because TCR γδ-chain rearrangement occurs before TCR αβ-chain rearrangement in the thymus, TCRs directed against self-antigens may be generated first. These Vγ2-chain T cells may therefore enter clonal anergy and prevent host immune activation against self-antigens. In addition, TCR αβ chains directed against self-antigens are eliminated during the positive and negative selection of CD4 and CD8 T cells, providing a second layer of protection. This mechanism resembles that of B cells co-expressing IgD and IgM. When self-antigens bind to IgD, B-cell IgM is not activated against self-antigens and therefore does not subsequently undergo isotype class switching to IgG, IgE, or IgA. In summary, this represents the proposed mechanism of Vγ2-chain γδ T-cell-mediated clonal anergy and tolerance [12,14]. Regarding the strength of the evidence supporting a role for Vγ2-chain γδ T cells in inducing clonal anergy, some evidence is available for this hypothesis. In a previous publication, we highlighted the association between γδ T cells and clonal anergy and summarized several relevant findings [13].

### 1.5. The Host Innate Immunological Pathway Against Viruses Is Associated with Vγ8-Chain γδ T Cells

The eradicable host adaptive immune response against viruses and prions is the THαβ immune reaction. In comparison with the antiviral THαβ immune reaction, the host innate immune response is mediated by Vγ8-chain γδ T cells. Notably, immune tolerance to self-antigens and innate immune reactions are both mediated by immune-cell subsets with limited diversity, including γδ T cells, invariant natural killer T cells (iNKT cells), and mucosa-associated invariant T cells (MAIT cells) [15,16,17,18,19,20]. Other important immune cells related to Vγ8-chain γδ T cells include natural killer cells (NK cells), invariant NKT10 cells (iNKT10), MAIT10 cells, and innate lymphoid cells (ILC10 cells) [21]. Many of these cells are invariant, consistent with limited antigen diversity. These cells can produce interleukin-10, a central mediator of antiviral immune responses. In addition, B-1a cells are immunoglobulin M (IgM)-producing B cells that provide antibodies for innate immune responses against viruses such as COVID-19, hepatitis C virus, and influenza virus [22,23,24]. These cell types are involved in host antiviral immune responses. Other infectious particles, such as prions, may also induce Vγ8-chain γδ T cells and related cell types. For example, CMV infection can induce activation of Vγ8-chain γδ T cells [25]. This innate immunological pathway is related to the THαβ immune reaction of eradicable immunity. The strength of evidence for the function of Vγ8-chain γδ T cells is moderate and less strong than the function of Vγ9-chain γδ T cells.

### 1.6. The Host Innate Immunological Pathway Against Intracellular Microorganisms Is Associated with Vγ9-Chain γδ T Cells

Vγ9-chain γδ T cells are γδ T-cell subtypes that mediate innate immune reactions against intracellular microorganisms, including intracellular bacteria. Other intracellular microorganisms, including intracellular fungi and protozoa, may also involve the activity of Vγ9-chain γδ T cells. In fact, γ9δ2 γδ T cells are the major γδ T-cell population in the blood circulation of both adults and fetuses [26]. Previous studies of intracellular pathogens involving innate γ9δ2 γδ T cells have reported immune reactions against Salmonella, Shigella, and other intracellular bacteria. These immune cells can expand dramatically and exceed all other lymphocyte populations within a few days in tuberculosis, salmonellosis, ehrlichiosis, brucellosis, tularemia, listeriosis, toxoplasmosis, and malaria. Other immune cells involved in this branch of the immunological pathway include iNKT1 cells, ILC1 cells, MAIT1 cells, and B1-b IgM-secreting B cells [27]. B1-b IgM B cells play an important role against intracellular microorganisms, including Toxoplasma, Borrelia, and Salmonella [28,29,30]. Macrophages are the main effector cells in innate immune reactions against intracellular microorganisms. All γ9δ2 γδ T cells can recognize the same small microbial compound, (E)-4-hydroxy-3-methyl-but-2-enyl phosphate (HMB-PP), a natural intermediate of the non-mevalonate pathway of isopentenyl pyrophosphate (IPP) biosynthesis [31]. HMB-PP is an essential metabolite in many pathogenic intracellular bacteria and protozoa, including *Mycobacterium tuberculosis* and malaria parasites, but is absent from host cells. Thus, HMB-PP may serve as a key antigenic target of γ9δ2 γδ T cells against intracellular bacteria, fungi, and protozoa [31,32]. This innate immunological pathway is related to the TH1 immune reaction of eradicable immunity [33]. Among all the γδ T cells, the strongest evidence are in Vγ9-chain γδ T cells. Vγ9-chain γδ T cells are the most abundant γδ T cells in the body and their functions against intracellular microorganisms including intracellular bacteria are well studied. In addition, molecular patterns of microorganism antigens are also identified for Vγ9-chain γδ T cells.

### 1.7. The Host Innate Immunological Pathway Against Extracellular Microorganisms Is Associated with Vγ4-Chain γδ T Cells

In extracellular bacterial infection, the host innate immune reaction is mediated mainly by Vγ4-chain γδ T cells. TRGV4 γδ T cells can produce interleukin-17, which is related to TH17 or TH22 adaptive immune responses. Other extracellular microorganisms, including extracellular fungi and protozoa, can also stimulate Vγ4-chain γδ T cells in host innate immunity. The major effector cells in this branch of host innate immunity are neutrophils, which perform phagocytosis to digest extracellular microorganisms. Other immune cells related to TRGV4 γδ T cells include iNKT17 cells, MAIT17 cells, ILC3 cells, and IgM-producing marginal zone B-1 cells (MZ B-1 cells) [21]. IgM-producing MZ B cells located in the splenic marginal zone are major B lymphocytes that encounter portal circulation from the intestine and produce natural IgM antibodies against extracellular bacteria, protozoa, and fungi from the gastrointestinal tract. iNKT17 cells, MAIT17 cells, and ILC3 cells can all produce interleukin-17 for immune responses against extracellular microorganisms [34]. This innate immunological pathway is related to the TH22 immune reaction of eradicable immunity. The strength of evidence for the function of Vγ4-chain γδ T cells is moderate and less strong than the function of Vγ9-chain γδ T cells.

### 1.8. The Host Innate Immunological Pathway Against Helminths Is Associated with Vγ5-Chain γδ T Cells

Host innate immunity against helminths is related to TRGV5 γδ T cells. TRGV5 γδ T cells are mainly located in the mucosa or skin. Notably, Vγ5 γδ T cells are also intestinal intraepithelial lymphocytes (IELs) [35,36,37]. Thus, TRGV5 γδ T cells can react to helminths (endoparasites), which mainly reside in the gastrointestinal or respiratory tract. TRGV5 γδ T cells are mucosa-associated γδ T cells. They can interact with Langerhans cells, which are involved in triggering TH2 immunity through antigen presentation. These cells may be related to intestinal allergy or skin atopy [38,39,40]. The principal effector cells in this innate immune pathway are mast cells and eosinophils. Other immune cells related to this innate immunological pathway include ILC2 cells, MAIT2 cells, iNKT2 cells, and IgM T2 cells [41,42,43]. This innate immunological pathway is related to the TH2a immune reaction of eradicable immunity. The strength of evidence for the function of Vγ5-chain γδ T cells is moderate and less strong than the function of Vγ9-chain γδ T cells.

### 1.9. The Host Innate Immunological Pathway Against Insects Is Associated with Vγ3-Chain γδ T Cells

**Figure 1:**
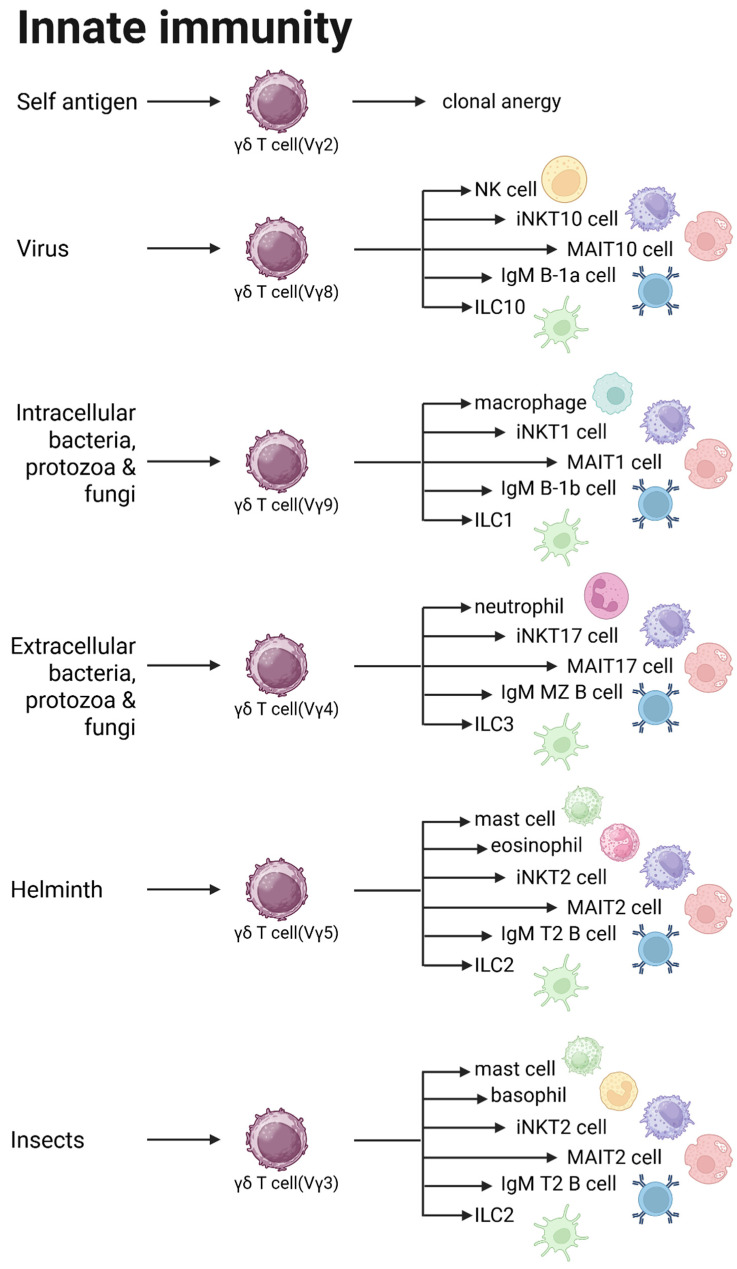
Framework of the host innate immunity pathway. The clonal anergy and tolerance pathway is associated with Vγ2-chain γδ T cells. The host innate immunological pathway against viruses is associated with Vγ8-chain γδ T cells. The pathway against intracellular microorganisms is associated with Vγ9-chain γδ T cells. The pathway against extracellular microorganisms is associated with Vγ4-chain γδ T cells. The pathway against helminths is associated with Vγ5-chain γδ T cells. The pathway against insects is associated with Vγ3-chain γδ T cells. In this figure, an arrow represents the triggering of specific immunological pathway. iNKT10 cells are interleukin-10-producing iNKT cells. MAIT10 cells are interleukin-10-producing MAIT cells. ILC10 cells are interleukin-10-producing innate lymphoid cells. IgM B1-a cells are IgM-producing B1-a cells. iNKT1 cells are type 1 iNKT cells. MAIT1 cells are type 1 MAIT cells. ILC1 cells are type 1 innate lymphoid cells. IgM B1-b cells are IgM-producing B1-b cells. iNKT17 cells are interleukin-17-producing iNKT cells. MAIT17 cells are interleukin-17-producing MAIT cells. ILC3 cells are type 3 innate lymphoid cells. IgM MZ B cells are IgM-producing marginal zone B cells. iNKT2 cells are type 2 iNKT cells. MAIT2 cells are type 2 MAIT cells. ILC2 cells are type 2 innate lymphoid cells. IgM T2 B cells are IgM-producing type 2 transitional B cells.

The innate immune reaction against insects is associated with Vγ3-chain γδ T cells. These TRGV3 γδ T cells are skin-resident cells in mice [44] and are also called dendritic epidermal T cells (DETCs) [35]. They can interact with Langerhans cells in the skin to trigger innate immune responses after insect bites. These insects are also called ectoparasites. One report indicated that TRGV3 cells are related to reactions to cockroach allergens [45]. These cells rapidly secrete interleukin-13 [46]. The main effector cells in this host innate immunological pathway are basophils and mast cells. Other immune cells related to this pathway include ILC2 cells, MAIT2 cells, iNKT2 cells, and IgM T2 B cells [46,47]. This innate immunological pathway is related to the TH2b immune reaction of eradicable immunity. The strength of evidence for the function of Vγ3-chain γδ T cells is moderate and less strong than the function of Vγ9-chain γδ T cells. In a previous publication, we highlighted the link of γδ T cells and clonal anergy and provided several research results [13]. In this article, we further discuss the association of γδ T cells and innate immune responses that was lacking in my previous publication. This review is more extensive. The framework of host innate immune pathways is presented in Figure 1.

The genetic sequence of these TRGV genes is TRGV2, TRGV3, TRGV4, TRGV5, TRGV8, and TRGV9, excluding pseudogenes such as TRGV6 and TRGV7. The proposed functions of these TRGV genes are as follows: TRGV2 is involved in T-cell clonal anergy; TRGV3 is involved in responses against insects; TRGV4 is involved in responses against extracellular bacteria, protozoa, and fungi; TRGV5 is involved in responses against helminths; TRGV8 is involved in responses against viruses; and TRGV9 is involved in responses against intracellular bacteria, protozoa, and fungi. It is noteworthy to compare the genetic order of these TRGV genes with the chromosomal order of B-cell antibody heavy-chain isotypes. The B-cell antibody heavy-chain isotype sequence is Cδ, Cγ3, Cγ1, Cγ2, Cγ4, and Cε. Cδ (IgD) is involved in B-cell clonal anergy. Cγ3 (IgG3) is involved in responses against intracellular bacteria, protozoa, and fungi; Cγ1 (IgG1) is involved in responses against viruses; Cγ2 (IgG2) is involved in responses against extracellular bacteria, protozoa, and fungi; Cγ4 (IgG4) is involved in responses against helminths; and Cε (IgE) is involved in responses against insects. Thus, the first position is also related to clonal anergy. Thereafter, the B-cell antibody heavy-chain isotype genetic order appears to show an inverse relationship to the γδ T-cell TRGV gene order, except for the order of TRGV4 and TRGV5. This exception may reflect the more urgent threat posed by insect venoms and extracellular bacteria [47]. Therefore, earlier genetic positioning may be advantageous for these responses. In addition, the pseudogenes TRGV6 and TRGV7 may help separate responses to extracellular and intracellular pathogens.

## 2. Adaptive Immunity

### 2.1. Framework of Host Adaptive Immunological Pathways

Host adaptive immune responses can be systematically classified into eradicable and tolerable pathways according to their functional objectives and regulatory mechanisms [13]. Eradicable host immune reactions are primarily initiated by follicular helper T cells (Tfh). These CD4^+^ T cells are characterized by the expression of the chemokine receptor CXCR5 and the secretion of interleukin-21 (IL-21). Activation of Tfh cells is regulated by the transcription factors BCL6 and STAT5B, which collectively promote the maturation of B cells within germinal centers. Through this interaction, Tfh cells induce antibody production and immunoglobulin class switching to IgG, thereby establishing highly effective humoral immunity against a wide range of pathogens.

### 2.2. The Host Adaptive Eradicable Immunological Pathway Against Intracellular Micro-Organisms Is Associated with TH1 Immune Response

Among eradicable pathways, the TH1 immune response is specialized for the elimination of intracellular microorganisms, including viruses and certain bacteria [14]. This pathway involves type 2 myeloid dendritic cells, type 1 innate lymphoid cells, M1-polarized macrophages, interferon-γ (IFN-γ)-secreting CD4^+^ T cells, cytotoxic CD8^+^ T cells (EM4, Tc1), type 2 natural killer T cells, and IgG3-producing B cells [15]. Interleukin-12 (IL-12) is the principal cytokine that drives TH1 differentiation. Effector M1 macrophages activated in this pathway destroy infected host cells through lipid membrane peroxidation and other free radical-mediated processes. Consequently, TH1 immunity is closely associated with type 4 delayed-type hypersensitivity reactions [48,49].

### 2.3. The Host Adaptive Eradicable Immunological Pathway Against Parasites Is Associated with TH2 Immune Response

The TH2 pathway is responsible for protective immunity against parasitic infections. It can be further divided into TH2a responses, which target endoparasites such as helminths, and TH2b responses, which act against ectoparasites. Each subtype engages distinct effector-cell populations and cytokine profiles, including eosinophils, basophils, mast cells, and IgE- or IgG4-producing B cells. Dysregulation of TH2 immunity contributes to type 1 immediate hypersensitivity and classical allergic diseases.

### 2.4. The Host Adaptive Eradicable Immunological Pathway Against Extracellular Micro-Organisms Is Associated with TH22 Immune Response

The TH22 pathway coordinates immunity against extracellular bacteria and fungi. Neutrophils (N1), IL-22-secreting CD4^+^ T cells, type 2 NKT cells, and IgG2-producing B cells participate in this response, which is mechanistically linked to type 3 immune complex-mediated hypersensitivity [50,51].

### 2.5. The Host Adaptive Eradicable Immunological Pathway Against Infectious Particles Is Associated with THαβ Immune Response

The THαβ pathway is specialized for the recognition and removal of infectious particles, including viruses and prions [52]. The key cytokines in the THαβ pathway are type 1 interferons and interleukin-10 [53]. Natural killer cells (NK1), IL-10-producing CD4^+^ T cells, cytotoxic CD8^+^ T cells (EM1, Tc2), and IgG1-producing B cells cooperate to eliminate pathogens, and this pathway is implicated in type 2 cytotoxic hypersensitivity reactions [54,55,56].

### 2.6. The Host Adaptive Tolerable Immunological Pathway Against Intracellular Micro-Organisms Is Associated with TH4 Immune Response

Tolerable host immune reactions are mediated by regulatory T cells (Tregs), which initiate immune modulation rather than direct destruction [57]. These cells express CD25 and secrete transforming growth factor-β (TGF-β), promoting antibody class switching to IgA through STAT5α and STAT5β activation. The TH4 pathway, previously termed the TH1-like pathway in our publication, represents a regulated form of TH1 immunity [58]. It responds to intracellular microorganisms through macrophages, IFN-γ/TGF-β-secreting CD4^+^ T cells, cytotoxic CD8^+^ T cells (EM3), type 2 NKT cells, and IgA1-producing B cells [59,60]. This pathway also contributes to type 4 hypersensitivity.

### 2.7. The Host Adaptive Tolerable Immunological Pathway Against Parasites Is Associated with TH9 Immune Response

The TH9 pathway addresses parasites under regulatory control and engages eosinophils (rEOS), basophils, mast cells, IL-9-secreting CD4^+^ T cells, type 2 NKT cells, and IgA2-producing B cells; it is likewise related to type 1 allergic hypersensitivity [61,62].

### 2.8. The Host Adaptive Tolerable Immunological Pathway Against Extracellular Micro-Organisms Is Associated with TH17 Immune Response

Furthermore, TH17 and TH3 pathways constitute major components of tolerable immunity against extracellular microorganisms and infectious particles, respectively. TH17 reactions involve IL-17-producing CD4^+^ T cells, neutrophils (N2), type 2 NKT cells, and IgA2-producing B cells and are associated with type 3 hypersensitivity [50].

### 2.9. The Host Adaptive Eradicable Immunological Pathway Against Infectious Particles Is Associated with TH3 Immune Response

**Figure 2:**
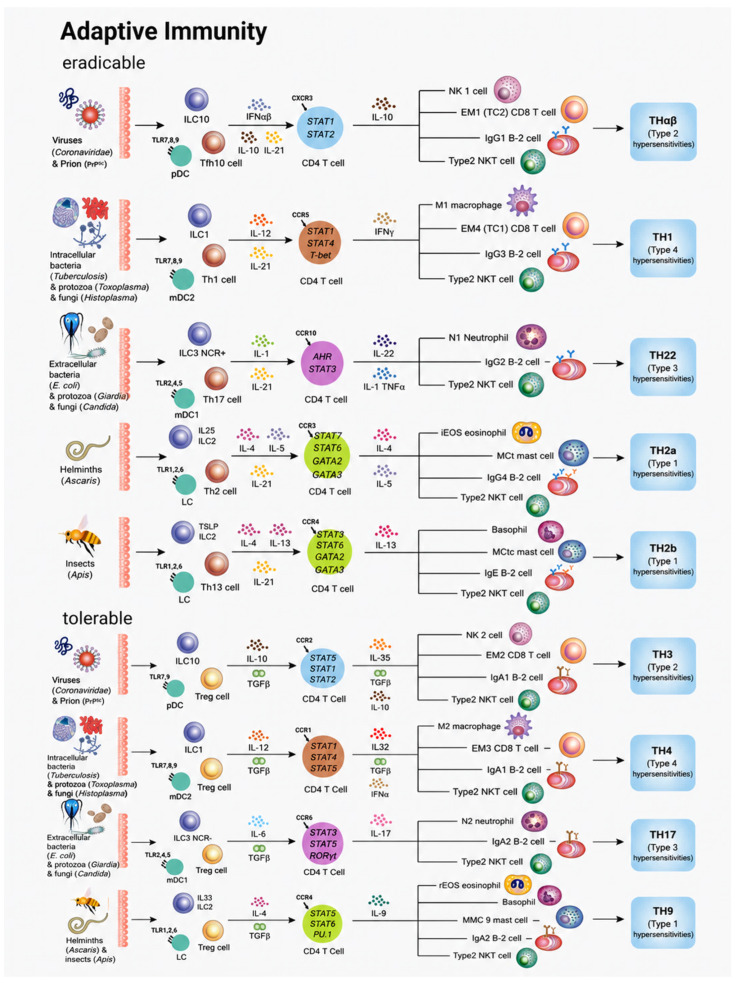
Framework of the host adaptive immunity pathway. Based on IgG or IgA responses, host adaptive immunity can be categorized as eradicable immunity or tolerable immunity. Eradicable host adaptive immunity includes TH1 (against intracellular microorganisms), TH2a (against helminths), TH2b (against insects), TH22 (against extracellular microorganisms), and THαβ (against infectious particles). Tolerable host adaptive immunity includes TH4 (against intracellular microorganisms), TH9 (against parasites), TH17 (against extracellular microorganisms), and TH3 (against infectious particles). This figure was modified from Wan-Chung Hu, Frontiers in Immunology, 2020, doi: 10.3389/fimmu.2020.01992 [11]. In this new modification, TH1-like immune response is renamed as TH4 immune response with interleukin-32-producing T cells are the major effector CD4 T cells. In addition, interleukin-35-producing CD4 T cells are assigned as the major CD4 T cells in TH3 immune response. These central cytokines play central roles in mediating TH4 or TH3 immune reactions. Their inclusion is therefore central to completing the framework. Thus, this is the key modification. And, the structure of innate immunological pathways and adaptive immunological pathways are comparable. In this graph, pDC denotes plasmacytoid dendritic cells, Tfh denotes the follicular helper T-cell types, mDC denotes myeloid dendritic cell types, TLR denotes Toll-like receptor, ILC denotes innate lymphoid cells, NK denotes natural killer cells, EM denotes effector-memory CD8 T cells, TC denotes cytotoxic T-cell types, NKT denotes natural killer T cells, LC denotes Langerhans cells, iEOS denotes inflammatory eosinophils, MCt denotes mast cells-trypsin, CCR/CXCR denotes chemokine receptor types, rEOS denotes regulatory eosinophils, Treg denotes regulatory T cells, MMC9 denotes interleukin-9-producing mucosal mast cells, STAT is the transcription factor of Signal Transducer and Activation of Transcription, AHR is the transcription factor of Aryl hydrocarbon receptor, PU.1 is the transcription factor of Spi-1 Proto-Oncogene, GATA denotes the transcription factor GATA binding protein.

The TH3 pathway is dominated by IL-10/TGF-β-producing regulatory CD4^+^ T cells, NK cells (NK2), CD8^+^ T cells (EM2), type 2 NKT cells, and IgA1-producing B cells and is implicated in type 2 cytotoxic hypersensitivity [55,63]. Notably, type 2 NKT cells can also be classified as NKT1, NKT2, NKT17, and NKT10 cells, analogous to type 1 NKT cells [64]. A schematic representation of this integrated framework of host immunological pathways is presented in Figure 2. The initial immune pathways were described in Frontiers in Immunology [11]. Subsequent modifications incorporated chemokine receptors, Toll-like receptors, and STAT-related transcription factors into the immune framework. TH2 immune responses were further divided into TH2a responses against helminths and TH2b responses against insects. In the present modification, the TH1-like immune response is renamed the TH4 immune response, with interleukin-32-producing T cells designated as the major effector CD4 T cells. In addition, interleukin-35-producing CD4 T cells are assigned as the principal CD4 T cells in the TH3 immune response. Below, we provide additional evidence supporting the designation of interleukin-32-producing T cells as the major effector TH4 CD4 T cells and interleukin-35-producing T cells as the major effector TH3 CD4 T cells.

### 2.10. Further Clarification of the TH3 and TH4 Immunological Pathways

TH3 and TH4 immune responses require clarification. TH3 is a well-known immunological reaction characterized by T cells that produce interleukin-10 and TGF-β [65]. However, interleukin-10 is the central cytokine of THαβ immunity, and TGF-β is the central cytokine of Treg cells. Therefore, another cytokine should represent the TH3 immunological pathway. Recently, a new subset of regulatory T cells distinct from interleukin-10-producing Tr1 cells and TGF-β-producing Treg cells was identified. These cells are called iTr35 cells and produce large amounts of interleukin-35 [66,67]. iTr35 cells can suppress TH1-, TH2-, and TH17-related cytokines but can upregulate interleukin-10, which is a key cytokine in THαβ immunity [67]. The TH3 immune reaction is the tolerable pathway corresponding to eradicable THαβ antiviral immunity [68,69]. Therefore, interleukin-35-secreting iTr35 cells are strong candidates for the key cellular component of TH3 immunity [68,70,71,72]. Indeed, TH3 conditions involving interleukin-10 and TGF-β can induce the production of interleukin-35. Interleukin-35 can also exert positive-feedback to increase interleukin-10 production. IL-35 can upregulate interleukin-10-producing Breg cells [73]. In addition, iTr35 cells have been identified as a stable clone of regulatory CD4 T cells [70,71,74]. Interleukin-35 can upregulate a regulatory NK-cell phenotype in tolerable immune responses [75]. These findings suggest that interleukin-35 and iTr35 cells are key components of the TH3 immune response [69,76,77]. Studies have also highlighted the important role of interleukin-35 in chronic viral infections, including hepatitis B, hepatitis C, EBV, RSV, and influenza virus infection [78,79,80,81]. Interleukin-35 is associated with EM2 cytotoxic T cells [82]. Thus, iTr35 cells may represent tolerable antiviral immune cells [67,81,83,84]. iTr35 cells are also related to tolerable immunity, including systemic sclerosis and suppression of collagen-induced arthritis [72,77,85]. iTr35 cells may therefore be considered TH3 cells. CD28− suppressive cytotoxic T cells (EM2 cytotoxic T cells) have been identified in systemic lupus erythematosus, which is a TH3-dominant autoimmune disease [82,86].

The TH4 immunological pathway was previously termed the TH1-like immune reaction in our publications. Here, we rename this pathway the TH4 immune pathway and identify the key cytokines associated with TH4 immunity. TH4 immunity is the tolerable counterpart of eradicable TH1 immunity and acts against intracellular microorganisms, including intracellular bacteria, protozoa, and fungi [87]. Macrophages are the key effector cells in TH1 and TH4 immunity against intracellular microorganisms. The central cytokine of TH4 immunity should be capable of shifting macrophages from an M1 to an M2 phenotype. In this review, we examined several cytokines induced by interleukin-12 together with TGF-β, including interleukin-32, interleukin-19, and platelet factor 4, which can promote M2 macrophage polarization [88,89]. Interleukin-32 is produced mainly by CD4 T cells and is associated with TH1 immunity [90]. Interleukin-32 has been implicated in host immunity against intracellular bacteria, protozoa, and fungi, including Chagas disease, tuberculosis, and Leishmania infection [87,91,92,93,94,95,96,97,98]. Interleukin-19 is produced mainly by monocytes or macrophages [99] and is associated with tolerable immune processes, such as fibrotic responses [100]. Platelet factor 4, also known as CXCL4, is produced mainly by platelets. Interleukin-37, an antagonist of interleukin-18, can also promote polarization toward the M2 macrophage phenotype. All of these cytokines can induce macrophage polarization from M1 to M2 phenotypes [89,101,102,103]. Platelet factor 4 is more appropriately classified as a chemokine than as a cytokine and also has regulatory functions [104]. Because the major cytokines of T-helper cells are produced predominantly by CD4 T cells, interleukin-32 may be the most representative cytokine of the TH4 immunological pathway [105]. Interleukin-32 functions as both an intracellular and extracellular cytokine [106]. Intracellular interleukin-32 may contribute to immunity against intracellular microorganisms [92]. Nevertheless, all of the cytokines and chemokines discussed above may be important components of TH4 immunity.

Once the key cytokines in TH3 and TH4 immunological pathways are identified, the framework of immunity can be completed. Some studies have reported that PF4 and interleukin-32 are related to a TH1-Treg immune reaction. This so-called TH1-Treg immune reaction is actually a TH4 immune response. Interleukin-32 can promote regulatory T cells as well as CD8 T cells, which is similar to a TH1-like condition [107]. Interleukin-2 and TCR stimulation can also stimulate interleukin-32 expression in T cells, suggesting that IL-32 is a regulatory T-cell-associated cytokine [105]. Interleukin-32 has also been reported in type 4 delayed-type hypersensitivities, which are TH1-dominant autoimmune disorders, including type 1 diabetes and granulomatosis with polyangiitis [108,109]. In addition, interleukin-32 is often produced by solid cancer cells [88,110]. In our previous publication, we proposed that TH1-like immunity (TH4 immunity) is a pro-tumor immunological pathway [13]. Moreover, interleukin-32 levels are associated with the prognosis of patients with cancer [111]. Thus, it is reasonable to propose that solid tumors can secrete interleukin-32 [107,110]. Based on the findings of this study, TH3 may also be called TH35, and TH4 may also be called TH32. In our previous publication, we proposed that CCR1 is the chemokine receptor of TH1-like immunity (TH4 immunity) [112]. Indeed, PF4 (CXCL4) is a chemokine ligand for CCR1 [113]. The main function of interleukin-37 is antagonism of interleukin-18, an enhancer of TH1 immunity. Therefore, interleukin-37 is unlikely to be the key cytokine of the TH4 immunological pathway. Interleukin-33, mainly produced by epithelial cells, induces TH2 immune responses and is therefore also not a strong candidate for TH4 immunity. TH3 and TH4 are preferable names in view of the history of immunology. In terms of pathogen defense, TH3 is related to tolerable anti-infectious-particle immunity, including responses to viruses and prions, whereas TH4 is related to tolerable anti-intracellular-microorganism immunity, including responses to intracellular bacteria, protozoa, and fungi. This framework may provide additional information and potential strategies for combating viruses and intracellular microorganisms. In the sections of eradicable immunological pathways, we propose the central cytokines including interleukin-32 for TH4 immunity and interleukin-35 for TH3 immunity. These cytokines have potent associations with TH3 or TH4 immunological pathways. Especially, interleukin-35-producing T cells, called iTr35 cells, are identified as tolerable immune cells suitable for my theory framework. The evidences are strong.

## 3. Cross-Talk Between Innate and Adaptive Immunity

### The Cross-Talk Between Innate Immunity and Adaptive Immunity

There is a cross-talk between innate immunity and adaptive immunity. γδ T cells can enhance the maturation of dendritic cells to up-regulate their functions of antigen presentations [114]. Both plasmacytoid dendritic cells and myeloid dendritic cells maturation and function can be triggered by γδ T cells [115,116]. On the other hand, the activated dendritic cells can further activate γδ T cells with a positive feedback loop [117]. The activation of dendritic cells can be the initial key step to activate the host adaptive immune reactions. In addition, γδ T cells can actually serve as antigen-presenting cells to prime CD8 T cells or typical CD4 T cells [118,119]. Antigen-activated γδ T cells can take and process foreign antigen to present it on MHC-II or cross-present on MHC-I molecule. γδ T cells activated with TCR and cytokines can up-regulate lymph node homing receptors, CD80/CD86/CD40 co-stimulator molecules to enter lymph modes to prime naïve CD4 or CD8 T cells. γδ T cells can also promote IgM production of B lymphocytes [120,121,122]. In addition, there is adaptive immunity-like trained immunity for γδ T cells. If host γδ T cells encountered certain pathogens before, they will produce larger amounts of cytokines including IFNg or interleukin-17 as well as chemokines like RANTES while they meet the certain pathogens again due to epigenetic reprogramming in γδ T cells [114]. For example, BCG vaccination can enhance the responsiveness of γδ T cells against *Mycobacterium tuberculosis*. This is the feedback from adaptive immune reactions to innate immune reactions.

## 4. Conclusions

Host innate immunological pathways can be categorized into six groups. The clonal anergy and tolerance pathway is associated with Vγ2-chain γδ T cells. The host innate immunological pathway against viruses is associated with Vγ8-chain γδ T cells. The pathway against intracellular microorganisms is associated with Vγ9-chain γδ T cells, whereas the pathway against extracellular microorganisms is associated with Vγ4-chain γδ T cells. The pathway against helminths is associated with Vγ5-chain γδ T cells, and the pathway against insects is associated with Vγ3-chain γδ T cells. Within adaptive immunity, five eradicable immune reactions and four tolerable immune reactions are described. Among the tolerable immune reactions, TH3 is associated with interleukin-35-producing CD4 T cells, and TH4 is associated with interleukin-32-producing CD4 T cells. A clearer understanding of the mechanisms underlying host innate and adaptive immune response pathways may support the development of additional strategies against infectious diseases and hypersensitivity reactions.

## Data Availability

No new data were created or analyzed in this study. Data sharing is not applicable to this article.

## Abbreviations

iNKT10 cells are interleukin-10-producing iNKT cells. MAIT10 cells are interleukin-10-producing MAIT cells. ILC10 cells are interleukin-10-producing innate lymphoid cells. IgM B1-a cells are IgM-producing B1-a cells. iNKT1 cells are type 1 iNKT cells. MAIT1 cells are type 1 MAIT cells. ILC1 cells are type 1 innate lymphoid cells. IgM B1-b cells are IgM-producing B1-b cells. iNKT17 cells are interleukin-17-producing iNKT cells. MAIT17 cells are interleukin-17-producing MAIT cells. ILC3 cells are type 3 innate lymphoid cells. IgM MZ B cells are IgM-producing marginal zone B cells. iNKT2 cells are type 2 iNKT cells. MAIT2 cells are type 2 MAIT cells. ILC2 cells are type 2 innate lymphoid cells. IgM T2 B cells are IgM-producing type 2 transitional B cells. pDC denotes plasmacytoid dendritic cells, Tfh denotes the follicular helper T-cell types, mDC denotes myeloid dendritic cell types, TLR denotes Toll-like receptor, ILC denotes innate lymphoid cells, NK denotes natural killer cells, EM denotes effector-memory CD8 T cells, TC denotes cytotoxic T-cell types, NKT denotes natural killer T cells, LC denotes Langerhans cells, iEOS denotes inflammatory eosinophils, MCt denotes mast cells-trypsin, CCR/CXCR denotes chemokine receptor types, rEOS denotes regulatory eosinophils, Treg denotes regulatory T cells, MMC9 denotes interleukin-9-producing mucosal mast cells, STAT is the transcription factor of Signal Transducer and Activation of Transcription, AHR is the transcription factor of Aryl hydrocarbon receptor, PU.1 is the transcription factor of Spi-1 Proto-Oncogene, GATA denotes the transcription factor GATA binding protein.
